# Fertility intentions to have a second or third child among the childbearing-age population in Central China under China’s three-child policy: A cross-sectional study

**DOI:** 10.7189/jogh.13.04072

**Published:** 2023-07-14

**Authors:** Qian Chen, Aihua Wang, Xinli Song, Xiaoying Liu, Yiping Liu, Jianhui Wei, Jing Shu, Mengting Sun, Taowei Zhong, Manjun Luo, Tingting Wang, Senmao Zhang, Donghua Xie, Jiabi Qin

**Affiliations:** 1 Department of Epidemiology and Health Statistics, Xiangya School of Public Health, Central South University, Changsha, Hunan, China; 2 Department of Information Management, Hunan Provincial Maternal and Child Health Care Hospital, Changsha, Hunan, China; 3 Department of Changsha Medical University Public Health Institute, Changsha, Hunan, China; 4 NHC Key Laboratory of Birth Defect for Research and Prevention, Hunan Provincial Maternal and Child Health Care Hospital, Changsha, Hunan, China; 5 Hunan Provincial Key Laboratory of Clinical Epidemiology, Changsha, Hunan, China

## Abstract

**Background:**

On May 31, 2021, the Chinese authorities announced that couples can have up to three children, aiming to stimulate a rise in fertility levels. However, there is limited research on second and third birth intentions of the childbearing-age population under China’s three-child policy, and the existing results are inconsistent.

**Methods:**

A cross-sectional survey was performed in Central China from June to August 2022. A total of 13 479 respondents aged 20-49 were enrolled in the study through a multi-stage sampling method. Data on the intentions to have a second or third child were collected using anonymized questionnaires. Descriptive statistics were performed to assess fertility intentions. Multivariate logistic regression analyses were used to assess the associations between fertility intentions and the related factors.

**Results:**

Among families with a single child, 29.7% (1444 / 4859) of the respondents intended to have a second child, while among two-child families, 10.6% (750 / 7056) respondents intended to have a third child. Overall, participants indicated that the ideal number of children was 1.85 ± 0.52. The age-specific fertility intentions of the one-child families were always higher than those of two-child families; however, based on couples’ age groups, the number of ideal children reported by two-child families was always higher than that of one-child families. Fertility intentions were influenced by the respondents’ gender, age, residence, marital status, educational level, average working time, childcare support, marital satisfaction, accessibility of educational resources, health condition of both spouses, loan situation, size of living house and the gender of the first child or second child.

**Conclusions:**

The general prevalence of the second and third birth intention of the childbearing-age population in Central China is not high. To increase the birth rate, it is necessary to create a favourable fertility context and offer supportive measures.

China introduced its one-child policy in 1979 [1]; the long-term implementation of this policy has resulted in imbalance of the sex ratio at birth, the shortage of labour resources, and severe population aging. Therefore, the Chinese government issued a conditional “two-child policy” in November 2013, permitting couples in which at least one of the partners was a single child to have two children, aiming to phase out the one-child policy and reverse the low fertility rate [2]; however, the implementation effect was lower than expected. Subsequently, the “universal two-child policy” was announced in October 2015, allowing all couples to have two children. Consequently, the fertility rate increased by 12.95‰ and 12.43‰ in 2016 and 2017, respectively [3]. However, the follow-up impetus of the policy was insufficient and its effect on fertility behaviour was limited. According to data from the seventh census [4], the number of births in mainland China in 2020 was only 12 million, the lowest in the country since 1962. Alarmingly, the census also revealed that China’s total fertility rate among women of childbearing age declined from 1.6 in 2017 to only 1.3 in 2020, which is lower than that of many developed countries, such as Japan (1.4) [5], the European Union (1.5), and the United States at (1.7) [5,6]. To improve China's population structure, actively respond to national population aging strategy, and maintain China's advantages of human resource endowment, on May 31, 2021, the authorities in China announced that couples can have up to three children and enjoy support measures [7]. However, it remains to be seen whether the policy will reverse the low fertility rate and help slow down population aging.

Fertility intentions are considered a major predictor of fertility behaviour [8]. A study in the United States revealed that although there are differences between fertility intentions and individual behaviour, there is relatively high consistency between aggregate intentions and actual fertility [9]. Therefore, fertility intentions can reliably predict aggregate human fertility behaviour which is of great significance to the study of fertility intentions. Currently, the declining trends in the natural growth and birth rates have not changed in Central China following the implementation of the universal two-child policy, with the downward trend increasing annually [10]. Low fertility rates are the result of multiple factors; one study showed that having completed an education, holding a job, a stable income, and house ownership are prominent factors for the making the decision of childbearing [11]. Hence, in addition to the announcement of the new three-child policy, the government is reportedly working on improving supportive measures that may promote couples to have more children and encourage births, covering various aspects of childbearing, childcare, parenting, education, taxation, housing, and women’s rights in employment [12]. In the new reforms, it is worth exploring whether the three-child policy is supported by the childbearing-age population, and it remains to be seen whether the supportive measures will actually stimulate people's fertility intentions. However, there is limited data and research on fertility intentions under the new policy. It is still unclear whether the new policy can prompt an increase in fertility.

Based on the above, we conducted a timely cross-sectional study in Hunan Province, Central China. The objectives of the study are: (1) to investigate fertility intentions among the childbearing-age population in Hunan Province under China’s three-child policy; (2) to assess factors influencing fertility intentions of the childbearing-age population after the enactment of China’s three-child policy; and (3) to determine the possible short-term effects of this policy on fertility patterns and provide recommendations for policy formulation.

## METHODS

### Study design and participants

We conducted a cross-sectional survey between June and August, 2022, by disseminating an online survey. A four-stage method was used to identify eligible participants. In the first stage, the thirteen cities and one autonomous prefecture in Hunan Province were divided into three regions (economically developed regions, economically medium regions, and economically underdeveloped regions) according to their gross domestic product (GDP) per capita ranking in 2021. From these three categories, the random number table method was adopted to randomly select an economically developed Changsha City, an economically medium Yueyang City and an economically underdeveloped Huaihua City. In the second stage, a simple random sample was used to select one county / city and one district in each of the three cities selected. In the third stage, towns / townships / streets / communities were selected by simple random sampling in the sampled county / city and district. In the fourth stage, study participants were selected using accidental sampling in the sampled towns / townships / streets / communities. The inclusion criteria for the participants were: (1) childbearing people aged between 20 and 49 years old, (2) married women and men, and (3) no history of severe physical or mental illnesses. Participants who were pregnant or had undergone permanent sterilization were not eligible for inclusion. The participants’ private information was strictly protected and data were anonymized. They had the right to refuse or quit the study at any point effective immediately.

The sample size was determined to establish the prevalence of the fertility intentions, assuming a prevalence of 37.0% in Central China based on a meta-analysis [13]. The formula n = *z*^2^*p* (1-*p*) / *d*^2^ for a cross-sectional study was used, where *n* is the minimum sample score for a normal distribution (*z* = 1.96), *p* is the presumed prevalence of fertility intentions (37.0%), and *d* is the margin of error (*d* = 0.01). Based on this formula, the minimum sample size was 8955. Because of the complexity of the error calculation process for multi-stage sampling, it was possible to multiply the existing sampling formula by a “design effect”. In this study, the sampling design effect *deff* was set to 1.2. In addition, a non-response rate of 20% was factored, which resulted in a sample size of 12 895. In this study, a total of 13 804 participants completed the questionnaires; of these, 312 participants who were out of the age range, and 13 who provided invalid data with anomalies or contradictions were excluded. Finally, 13 479 participants were included in the analysis. We used the Wen Juan Xing platform for the questionnaire dissemination because it is the largest online survey platform in China, covering over 2.6 million respondents. Web-based questionnaires were sent to the selected individuals who agreed to participate in the survey, and participants completed the questionnaires online using mobile phones.

### Definition of fertility intention

Fertility intention refers to people’s desire to have children and pursue childbirth [14], and is impacted by the expectations of the number, timing, gender, and quality of the children. Fertility intention-the dependent variable and primary outcome-was assessed by the following question: “Do you intend to have another child under China’s three-child policy?” with the response options being: “No” or “Yes”.

### Procedure

The Chinese language version of the questionnaire was developed by experts in the field of demography research, and administered to eligible participants (test-retest reliability = 0.823; Cronbach’s alpha = 0.785). The questionnaire including sociodemographic characteristics (eg, age, gender, ethnicity, residence, marital status, education level, average working hours, mobile population and insurance), family status (parent’s family situation, whether living with parents, childcare support, relationship between mother-in-law and daughter-in-law, marital satisfaction, educational and medical barriers for the children), family members' health status (spouses, parents, and child), family economic situation (monthly income, loan situation, size of living house and parents' financial situation), gender pattern and fertility intentions. All survey items needed to be answered before the questionnaire could be submitted to ensure the effectiveness of data collection. Participants were assigned identification numbers, which could be used only once. After data collection, the questionnaire responses were collated and examined by the research team.

### Statistical analysis

The results are presented in tables, charts and graphs, as appropriate. Basic analyses were performed using SPSS 26.0 software (IBM SPSS Inc, Chicago, USA). Graphs were prepared in Graphpad Prism, version 8.0.1 (GraphPad Software Inc, San Diego, USA) and forest plots were conducted using R software, version 3.5.0 (Robert Gentleman and Ross Ihaka, Aucklandz Auckland, New Zealand). Qualitative data were described using frequencies and percentages, and quantitative data were described using means and standard deviations (SD). The prevalence of fertility intention to have another child was calculated, and between-group comparisons were performed using the χ^2^ test or the non-parametric Wilcoxon rank sum test, as appropriate. A binary logistic regression with “the fertility intentions to have another child (No or Yes).” as the dependent variable and the investigated factors as explanatory variables was conducted to test the associations between them. According to the univariate analysis results, covariables with *P* < 0.10 were included in the multivariate logistic regression model. Using the stepwise backward elimination method, the variables that retained significance (*P* < 0.05) were maintained in the final multivariate model. Multivariate logistic regression analysis was conducted to explore the potential associated factors, with results presented as odds ratio (OR), 95% confidence interval (95% CI) and *P*-value. In this study, the number of childless families and three-child families was small, therefore, these groups were not included in the univariate and multivariate logistic regression analysis.

### Ethics approval

This study was based on data from a cross-sectional study on the childbearing population in the Hunan Province. This study received ethics approval from the Hunan Provincial Maternal and Child Health Care Hospital. A cover letter was presented and all participants were provided an explanation of the study purpose and procedures. All participants recruited in this study asserted their consent to participate by choosing the “I agree” option ahead of filling in the questionnaires. Participants were also informed that they could withdraw from the study at any time and could contact the investigators if they had any questions about the questionnaire. If the participants could not answer the questionnaires online, the specially trained investigators and their teams were to provide a one-to-one interview by means of written and verbal communication. The participants’ responses were kept confidential and were only accessible to the research team.

## RESULTS

### Basic characteristics and fertility intentions of the study population

**Figure 1:**
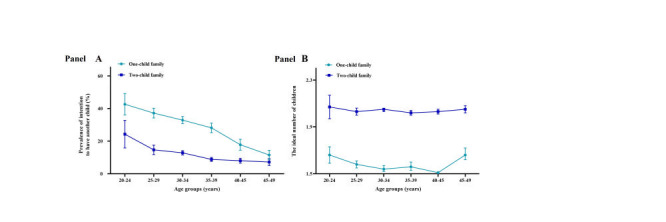
Age-specific fertility intentions by family type. **Panel A.** Prevalence of fertility intentions to have another child by family type. **Panel B.** Mean ideal number of children by family type. Light blue symbols show one-child family, and dark blue symbols show two-child family.

During the recruitment phase, a total of 13 479 respondents from Hunan Province were enrolled in the study, including 892 (6.6%) respondents with no children, 4859 (36.1%) with one child, 7056 (52.3%) with two children and 672 (5.0%) with three children. The prevalence of fertility intentions to have another child are shown in **Table 1**. Among childless families, 35.9% (320 / 892) respondents intended to have their first child. In one-child families, 29.7% (1444 / 4859) of the respondents intended to have a second child. In two-child families, 10.6% (750 / 7056) of the respondents intended to have a third child. In three-child families, 14.4% (97 / 672) of the respondents intended to have another child. Overall, participants indicated that the ideal number of children was 1.85 ± 0.56. The prevalence of age-specific fertility intentions by family type are summarized in Table S1 in the **Online Supplementary Document**. In total, the age-specific fertility intentions of the one-child families were always higher than those of the two-child families (**Figure 1**, panel A). For both one-child and two-child families, the intention to have another child decreased with age; the highest intention to have another child was observed among couples aged 20-24 years (**Figure 1**, panel A). Furthermore, for age groups, he ideal number of children in the two-child families was always higher than that in the one-child families (**Figure 1**, panel B). For the one-child families, the ideal number of children was between 1.5 and 1.7 in all age groups. For the two-child families, the ideal number of children was closer to 2 at all ages (**Figure 1**, panel B).

**Table 1:** The overall intention for the number of children among study respondents

	n	%
**Intention for a first child (n = 892)**		
Yes	320	35.9
No	572	64.1
**Intention for a second child (n = 4589)**		
Yes	1444	29.7
No	3415	70.3
**Intention for a third child (n = 7056)**		
Yes	750	10.6
No	6306	89.4
**Intention for a fourth child (n = 672)**		
Yes	97	14.4
No	575	85.6
**The ideal number of children among all respondents (n = 13479)**		
0	137	1.0
1	2839	21.1
2	9370	69.5
≥3	1133	8.4
**Mean ± SD**	1.85 ± 0.56	

SD – standard deviation

### Univariate analysis

The sociodemographic characteristics of the one-child and two-child families are shown in **Table 2**. For the one-child families, the unadjusted analysis showed that the respondents’ gender, age, residence, marital status, educational level, average working hours, and floating population were associated with fertility intentions. For the two-child families, the respondents’ gender, age, residence, marital status, educational level, average working hours and insurance were associated with fertility intentions.

**Table 2:** Univariate analysis of the influence of sociodemographic characteristics on fertility intention

Factors	One child family fertility intention			Two child family fertility intention		
	**Yes**	**No**	***P*-value**	**Yes**	**No**	***P*-value**
The gender of the respondents			0.037*			0.011*
*Women*	1357 (29.4)	3258 (70.6)		701 (10.4)	6025 (89.6)	
*Men*	87 (35.7)	157 (64.3)		49 (14.8)	281 (85.2)	
Age (years)			<0.001‡			<0.001‡
*20-24*	95 (42.6)	128 (57.4)		25 (24.3)	78 (75.7)	
*25-29*	374 (37.2)	632 (62.8)		86 (14.7)	500 (85.3)	
*30-34*	578 (32.9)	1180 (67.1)		311 (12.8)	2111 (87.2)	
*35-39*	254 (28.1)	649 (71.9)		187 (8.8)	1939 (91.2)	
*40-44*	89 (17.8)	411 (82.2)		100 (8.0)	1149 (92.0)	
*45-49*	54 (11.5)	415 (88.5)		41 (7.2)	529 (92.8)	
Ethnicity			0.198			0.330
*Han*	1330 (29.5)	3181 (70.5)		696 (10.5)	5910 (89.5)	
*Minority*	114 (32.8)	234 (67.2)		54 (12.0)	396 (88.0)	
Residence			<0.001‡			<0.001‡
*Urban*	509 (23.3)	1672 (76.7)		179 (7.9)	2098 (92.1)	
*Rural*	935 (34.9)	1743 (65.1)		571 (11.9)	4208 (88.1)	
Marital status			0.032*			<0.001‡
*First marriage*	1414 (29.5)	3372 (70.5)		710 (10.3)	6215 (89.7)	
*Remarriage*	30 (41.1)	43 (58.9)		40 (30.5)	91 (69.5)	
Educational level			<0.001‡			<0.001‡
*Junior high school or below*	203 (38.3)	327 (61.7)		232 (13.7)	1460 (86.3)	
*Senior high school or equivalent*	303 (31.7)	653 (68.3)		243 (12.0)	1778 (88.0)	
*College or higher*	938 (27.8)	2435 (72.2)		275 (8.2)	3068 (91.8)	
Average working hours			<0.001‡			<0.001‡
*<7 h*	518 (35.1)	956(64.9)		341 (13.2)	2251 (86.8)	
*7-12 h*	886 (27.6)	2327(72.4)		377 (9.1)	3768 (90.9)	
*>12 h*	40 (23.3)	132(76.7)		32 (10.0)	287 (90.0)	
Floating population			0.003†			0.191
*No*	495 (32.7)	1021(67.3)		529(10.3)	4590 (89.7)	
*Yes*	949 (28.4)	2394(71.6)		221(11.4)	1716 (88.6)	
Insurance			0.239			0.002†
*No*	21 (25.6)	61 (74.4)		27 (18.5)	119 (81.5)	
*Yes*	1423 (29.8)	3354 (70.2)		723 (10.5)	6187 (89.5)	

*P < 0.05.

†*P* < 0.01.

‡*P* < 0.001.

The findings of the family status associated with fertility intentions are shown in **Table 3**. For the one-child families, the unadjusted analysis showed that living with parents, childcare support, relationship between mother-in-law and daughter-in-law, marital satisfaction, and educational and medical barriers for children were associated with fertility intentions. For the two-child families, in addition to the factors mentioned above, parents' family situation was related to fertility intentions.

**Table 3:** Univariate analysis of the influence of family status on fertility intention

Factors	One child family fertility intention			Two child family fertility intention		
**Yes**	**No**	***P*-value**	**Yes**	**No**	***P*-value**
Parent’s family situation			0.840			0.019‡
*Both only child*	141 (29.4)	339 (70.6)		16 (6.8)	221 (93.2)	
*Both non-only child*	892 (30.0)	2079 (70.0)		609 (11.1)	4859 (88.9)	
*One of only child*	411 (29.2)	997 (70.8)		125 (9.3)	1226 (90.7)	
Living with parents			<0.001‖			0.006§
*No*	514 (24.3)	1602 (75.7)		503 (11.4)	3907 (88.6)	
*Yes*	930 (33.9)	1813 (66.1)		247 (9.3)	2399 (90.7)	
Childcare support			<0.001‖			<0.001‖
*No*	375 (19.9)	1511 (80.1)		234 (8.1)	2665 (91.9)	
*Yes*	1069 (36.0)	1904 (64.0)		516 (12.4)	3641 (87.6)	
Relationship between mother-in-law and daughter-in-law			<0.001‖			0.002§
*Bad*	23 (14.4)	137 (85.6)		12 (6.3)	179 (93.7)	
*Ordinary*	352 (22.5)	1209 (77.5)		200 (9.1)	1989 (90.9)	
*Good*	1069 (34.1)	2069 (65.9)		538 (11.5)	4138 (88.5)	
Marital satisfaction			<0.001‖			<0.001‖
*Dissatisfied*	56 (15.4)	308 (84.6)		20 (3.9)	492 (96.1)	
*Satisfied*	1388 (30.9)	3107 (69.1)		730 (11.2)	5814 (88.8)	
Children’s educational barriers*			<0.001‖			<0.001‖
*Yes*	206 (23.8)	660 (76.2)		105 (7.8)	1244 (92.2)	
*No*	1238 (31.0)	2755 (69.0)		645 (11.3)	5062 (88.7)	
Children’s medical barriers**†**			<0.001‖			<0.001‖
*Yes*	198 (24.1)	622 (75.9)		96 (7.9)	1113 (92.1)	
*No*	1246 (30.8)	2793 (69.2)		654 (11.2)	5193 (88.8)	

*Children’s educational barriers means the stress of the accessibility of educational resources and meet the educational needs of children.

†Children’s medical barriers means the stress of the accessibility of medical resources and meet the health needs of children.

‡*P* < 0.05.

§*P* < 0.01.

‖*P* < 0.001.

Data for the participants’ family members' health status are shown in **Table 4**. For the one-child families, the unadjusted analysis showed that the health condition of both spouses and both parents was associated with fertility intentions. For the two-child families, the health condition of both spouses was related to fertility intentions.

**Table 4:** Univariate analysis of the influence of family members' health status on fertility intention

Factors	One child family fertility intention			Two child family fertility intention		
**Yes**	**No**	***P*-value**	**Yes**	**No**	***P*-value**
Health condition of both spouses			<0.001*			<0.001*
*Both healthy*	1264 (31.5)	2750 (68.5)		671 (11.4)	5220 (88.6)	
*Both unhealthy*	75 (18.0)	342 (82.0)		45 (7.2)	578 (92.8)	
*One of only healthy*	105 (24.5)	323 (75.5)		34 (6.3)	508 (93.7)	
Health condition of both parents			<0.001*			0.236
*Both healthy*	1367 (30.7)	3083 (69.3)		695 (10.8)	5276 (89.2)	
*Both unhealthy*	10 (19.6)	41 (80.4)		9 (9.3)	88 (90.7)	
*One of only healthy*	67 (18.7)	291 (81.3)		46 (8.6)	492 (91.4)	
Health condition of child			0.202			0.808
*Both healthy*	1419 (29.8)	3336 (70.2)		711 (10.6)	5991 (89.4)	
*≥1 unhealthy*	25 (24.0)	79 (76.0)		39 (11.0)	315 (89.0)	

*P < 0.001.

The unadjusted analysis of the association between family economic situation and fertility intentions (**Table 5**) showed that the loan situation, size of living house, and parents' financial situation were the relevant factors in the one-child and two-child families.

**Table 5:** Univariate analysis of the influence of family economic situation on fertility intention

Factors	One child family fertility intention			Two child family fertility intention		
**Yes**	**No**	***P*-value**	**Yes**	**No**	***P*-value**
Monthly income (RMB)			0.192			0.278
*<5000*	2347 (69.4)	1035 (30.6)		598 (11.0)	4862 (89.0)	
*5000-10 000*	345 (27.6)	907 (72.4)		132 (9.8)	1218 (90.2)	
*10 001-20 000*	46 (27.2)	123 (72.8)		12 (7.1)	157 (92.9)	
*>20 000*	18 (32.1)	38 (67.9)		8 (10.4)	69 (89.6)	
Loan situation			<0.001*			<0.001*
*Yes*	852 (27.7)	2221 (72.3)		385 (9.4)	3709 (90.6)	
*No*	592 (33.1)	1194 (66.9)		365 (12.3)	2597 (87.7)	
Size of living house			<0.001*			<0.001*
*<100 m^2^*	522 (26.6)	1444 (73.4)		217 (9.4)	2087 (90.6)	
*100-200 m^2^*	861 (31.2)	1900 (68.8)		478 (10.8)	3951 (89.2)	
*>200 m^2^*	61 (46.2)	71 (53.8)		55 (17.0)	268 (83.0)	
Parents' financial situation			<0.001*			<0.001*
*Poor*	230 (23.8)	737 (76.2)		147 (8.4)	1612 (91.6)	
*Rich*	1214 (31.2)	2678 (68.8)		603 (11.4)	4694 (88.6)	

*P < 0.001.

Additionally, univariate analysis (**Table 6**) showed that the gender pattern of families with one or two children were significantly different between the two groups with fertility intentions and without fertility intentions.

**Table 6:** Fertility intentions and gender pattern of one child and two child families

Gender pattern	Fertility intention		
**Yes**	**No**	***P*-value**
The gender pattern of one child family			<0.001*
*Boy*	752 (27.6)	1971 (72.4)	
*Girl*	692 (32.4)	1444 (67.6)	
The gender pattern of two-child family			<0.001*
*Girl and boy*	170 (8.0)	1946 (92.0)	
*Boy and boy*	182 (10.1)	1626 (89.9)	
*Boy and girl*	132 (7.7)	1580 (92.3)	
*Girl and girl*	266 (18.7)	1154 (81.3)	

*P < 0.001.

### Multivariable analysis

Multivariate logistic regression by parity (having either one or two children) was performed to identify the factors associated with fertility intention to have another child (Yes or No) (Table S2 and Table S3 in the **Online Supplementary Document**).

**Figure 2:**
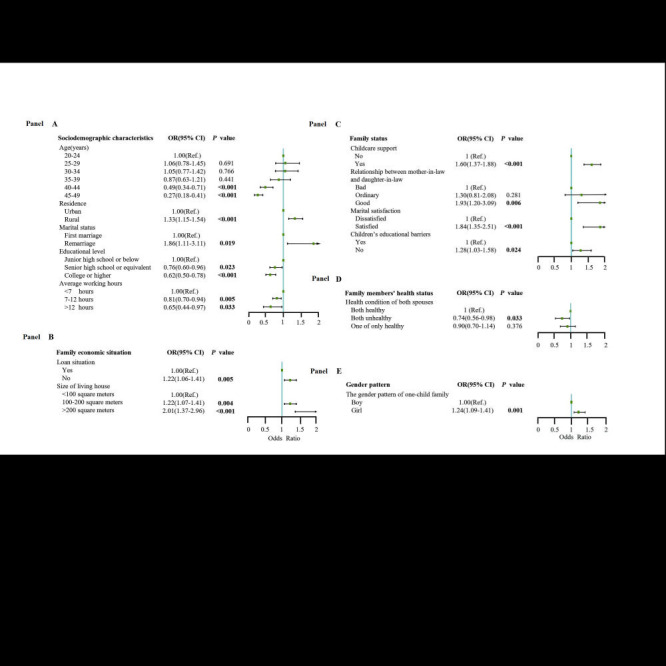
The one-child family multivariate analysis.

For the one-child families, we further conduced multivariate analysis with mutual adjustment among the significant factors in the univariate analysis (**Figure 2**). The results showed that living in rural areas (adjusted odds ratio (aOR) = 1.33, 95% CI = 1.15-1.54), remarriage (aOR = 1.66, 95% CI = 1.11-3.11), having childcare support (aOR = 1.60, 95% CI = 1.37-1.88), having good relationship between mother-in-law and daughter-in-law (aOR = 1.93, 95% CI = 1.20-3.09), having satisfied marriage (aOR = 1.84, 95% CI = 1.35-2.51), having convenient educational resources (aOR = 1.28, 95% CI = 1.03-1.58), having 101-200 m^2^ living house (aOR = 1.22, 95% CI = 1.07-1.41), having more than 200 m^2^ living house (aOR = 2.01, 95% CI = 1.37-2.96) and having a girl (aOR = 1.24, 95% CI = 1.09-1.41) were associated with a higher intention to have a second child (all *P* < 0.05). Additionally, 40-44 years old (aOR = 0.49, 95% CI = 0.34-0.71), 45-49 years old (aOR = 0.27, 95% CI = 0.18-0.41), senior high school or equivalent (aOR = 0.76, 95% CI = 0.60-0.96), college or higher (aOR = 0.63, 95% CI = 0.51-0.79), having 7-12 hours working time (aOR = 0.81, 95% CI = 0.70-0.94), having more than 12 hours working time (aOR = 0.65, 95% CI = 0.44-0.97) and spouses both unhealthy (aOR = 0.74, 95% CI = 0.56-0.98) were associated with a lower intention to have a second child (all *P* < 0.05).

**Figure 3:**
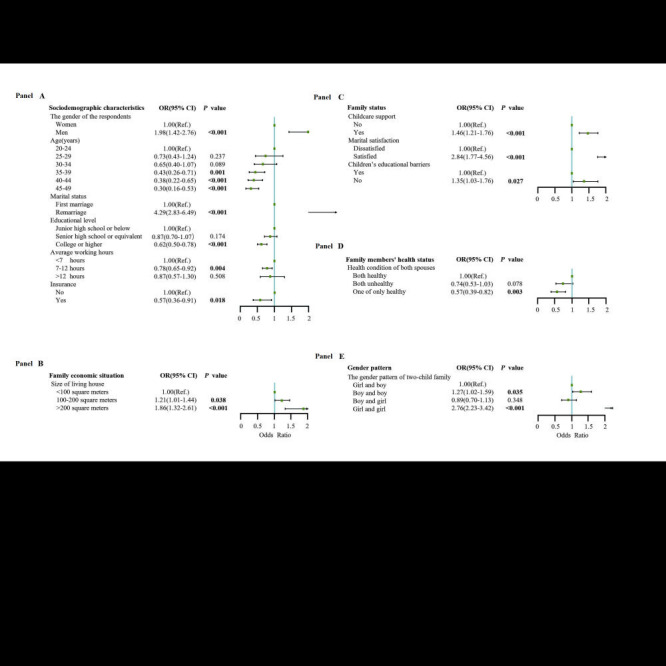
The two-child family multivariate analysis.

For the two-child families, we further conduced multivariate analysis with mutual adjustment among these significant factors in univariate analysis (**Figure 3**), showed that the men respondents (aOR = 1.98, 95% CI = 1.42-2.76), remarriage (aOR = 4.29, 95% CI = 2.83-6.49), having childcare support (aOR = 1.46, 95% CI = 1.21-1.76), having satisfied marriage (aOR = 2.84, 95% CI = 1.77-4.56), having convenient educational resources (aOR = 1.35, 95% CI = 1.03-1.76), having 101-200 m^2^ living house(aOR = 1.21, 95% CI = 1.01-1.44), having more than 201 m^2^ living house (aOR = 1.86, 95% CI = 1.32-2.61), having two boys (aOR = 1.27, 95% CI = 1.02-1.59) and having two girls (aOR = 2.76, 95% CI = 2.23-3.42) were associated with a higher intention to have a third child (all *P* < 0.05), additionally, 35-39 years old (aOR = 0.43, 95% CI = 0.26-0.71), 40-44 years old (aOR = 0.38, 95% CI = 0.22-0.65), 45-49 years old (aOR = 0.30, 95% CI = 0.16-0.53), college or higher (aOR = 0.62, 95% CI = 0.50-0.78), having 7-12 hours working time (aOR = 0.78, 95% CI = 0.65-0.92), participate in insurance (aOR = 0.57, 95% CI = 0.36-0.91), and spouses one of only healthy (aOR = 0.56, 95% CI = 0.39-0.81) were associated with a lower intention to have a third child (all *P* < 0.05).

## DISCUSSION

To the best of our knowledge, this is the first cross-sectional study to comprehensively asses the fertility intentions and associated factors among the population of childbearing age in Central China after the implementation of the three-child policy. In the context of an overall low fertility rate, the intention rate among one-child families for second births was 29.7% and that among two-child families for a third child was 10.6% in Central China, which was inconsistent with previous surveys [15-17]. The possible reasons for the differences are that fertility intentions are influenced by the duration of birth policy implementation and the characteristics of different populations. Some scholars have found that although China’s population policy is gradually opening up, the ideal number of children has been declining in recent years. Furthermore, economic development, improvement in education levels and the COVID-19 pandemic may lead to a continuous decline in the ideal number of children [18,19]. While most one-child families would like to have an ideal number of two children, financial, educational and other factors prevent them from having a second child; whereas, for families with two children, the ideal number of children is closer to two, indicating a match between their ideal and actual number of children [16].

Previous studies demonstrated that the fertility intentions of women were lower than those of men [20, 21]; our study obtained similar results in two-child families. This may be related to the diversity in social responsibilities owing to gender differences. Regarding reproductive behaviour, women bear the primary responsibility for pregnancy and childbirth, which poses greater risks to their physical and mental health compared with men. Various evidence has shown that women bearing more children implies greater challenges and threats to job stability and career development [22-24], while men perceive fewer problems relating to balancing work and family life [25]. Therefore, women are more cautious and reserved in expressing their desire to have more children. Hence, child-rearing policies and supportive measures should be improved to help women manage work and childcare and encourage fathers to participate in child-rearing.

We found that the participants’ intentions to have a second and third child gradually declined with an increasing age. Several studies have confirmed that the optimal reproductive age for women is before the age of 35 [26], and older age can lead to infertility and increased pregnancy complications [14]. Hence, if the government provides regular pre-pregnancy health promotion for older couples in primary health care facilities, helping them believe that the risks of pregnancy are better controlled, it may improve their willingness to have a second and third child. Additionally, we found that participants who remarried were more likely to have a second and third child, compared with those in their first marriage; this is consistent with the findings of Wu and Zhang [17,27]. The possible reasons may be related to the tendency to choose co-fertility in reorganized families, which could help maintain and strengthen emotional relationships between couples and family members [15,17]. Regarding the regions, evidence from the literature [15,17] illustrates that the fertility intentions of the rural population are higher than the urban population, which is consistent with our results. The reasons may be associated with the different regional contexts and fertility ideologies [28], ascribed to lower parenting costs in rural areas that make it easier to raise a child compared with urban areas.

Our results showed that respondents with higher education and longer working hours, and couples with at least one unhealthy partner had a lower intention to have a second or third child. Evidence from other developing countries emphasizes that women’s fertility intentions tend to decline as they obtain higher education [29], which may be related to the phenomenon that higher education allows women to pursue their own careers and be free of raising children [30]. In addition, raising, caring for, and educating children consumes time and energy. Regardless of the occupation of the childbearing-age population, a busy work schedule, combined with raising multiple children, can exhaust parents. This is also the main reason why less disposable time decreases a family’s desire to have children [31]. Therefore, if a couple's parents can provide childcare support, they may have higher intention to have a second and third child. Our results corroborate this idea. Moreover, a previous study found that participants who were more concerned about their own health and that of their unborn child were more likely to terminate their pregnancy [32], possibly because the couple’s health condition may affect the health of the unborn child or because the couple may not be able to take good care of the child if they are not in good health themselves.

Reproductive decisions are based on couples’ intentions; recent studies in the United States confirmed this view, showing that couples’ marital relationship affects the likelihood of having children [33,34]. Further, Mencarini et al. [35] emphasized that life satisfaction is conducive to the improvement of fertility intention in countries or regions with low fertility rates, which is in line with the presents results. In addition, the family’s economic status was found to be associated with fertility intentions [36]. Greater economic resources mean that families can afford the high costs of raising children and obtain convenient educational resources; this is also in accordance with additional observations in the present study.

The gender pattern is another a factor affecting fertility intentions. First, since ancient times, there has been a traditional preference in China for sons over daughters especially in rural areas [37]. Further, the cost of marriage for girls is generally lower than that for boys in China [15]. In addition, China’s population policy is gradually opening up. Thus, couples with either one or two daughters do not yet have a high financial burden, and are therefore more likely to follow the long-standing preference for having a boy. Additionally, couples with two sons may want to have a daughter as well, and may have higher intention to have a third child.

Although the three-child policy has been introduced, relying solely on the policy to effectively alleviate the declining fertility trend is insufficient. Policy makers need to introduce supportive measures, such as providing maternity subsidies, increasing maternity leave, increasing financial investment in public nursery services, and reducing the cost of children’s education. On the basis of giving full play to the leading role of the government and strengthening awareness of gender equality in society, it is recommended that barriers to women's employment should be removed and a universal childcare service vigorously developed, so as to alleviate the work-family conflict among women and improve the effect of fertility support policies. Without wide-ranging supportive measures aimed at encouraging childbirth, the country may face a continuous decline in fertility rates in the coming decades.

### Limitations

There are some limitations to our study. First, as this was a single-centre study in China, the results may not be applicable to other provinces. Moreover, we applied a multi-stage sampling method for this survey and selection bias could not be eludible. Future research should focus on people from different regions of China. Second, as this study used the Wen Juan Xing platform to distribute the questionnaires, response bias could not be estimated. Third, as our participants only included married couples and we did not assess the fertility intentions of the unmarried population, a fraction of possible error owing to non-marital births could not be avoided. In addition, while this study reflects the fertility intentions of couples of childbearing age in Hunan province, providing valuable data for government policy makers, it could not determine causal relationships in the associations between the variables.

## CONCLUSION

This study assessed the impact of the three-child policy on the fertility intentions of the population of childbearing age in Hunan Province. The general prevalence of the second and third birth intention in the childbearing-age population in Hunan province is not high. The decision to have more children was found to be influenced by the respondents’ gender, age, residence, marital status, educational level, average working time, childcare support, marital satisfaction, accessibility of educational resources, health condition of both spouses, loan situation, size of living house, and the first or second child’s gender. It is necessary to create a favourable fertility context and provide supportive measures for the childbearing-age group to increase fertility intentions.

## Additional material


Online Supplementary Document

## References

[1] Hesketh T Lu L Xing ZW The effect of China’s one-child family policy after 25 years. N Engl J Med. 2005;353:1171-6. 10.1056/NEJMhpr051833 16162890

[2] Feng W Gu B Cai Y The End of China’s One-Child Policy. Stud Fam Plann. 2016;47:83-6. 10.1111/j.1728-4465.2016.00052.x 27027994

[3] Council PCOotS. China's fertility rate drops to low level 2022. Available: http://www.scio.gov.cn/xwfbh/xwbfbh/wqfbh/44687/46355/xgbd46362/Document/1709084/1709084.htm. Accessed: 1 November 2022.

[4] Statistics NBo. Main Data of the Seventh National Population Census. 2021. Available: http://www.stats.gov.cn/english/PressRelease/202105/t20210510_1817185.html. Accessed: 10 November 2022.

[5] Review WP. Total Fertility Rate 2022 2022. Available: https://worldpopulationreview.com/country-rankings/total-fertility-rate. Accessed: 5 November 2022.

[6] Times G. China’s fertility rate may become ‘world’s lowest’ without strong intervention policy, India may overtake China by 2023: demographers. 2022. Available: https://www.globaltimes.cn/page/202105/1223141.shtml. Accessed: 8 November 2022.

[7] Tatum M China’s three-child policy. Lancet. 2021;397:2238. 10.1016/S0140-6736(21)01295-2 34119051

[8] Lau BH Huo R Wang K Shi L Li R Mu S Intention of having a second child among infertile and fertile women attending outpatient gynecology clinics in three major cities in China: a cross-sectional study. Hum Reprod Open. 2018;2018:hoy014. 10.1093/hropen/hoy014 30895255 PMC6276692

[9] Quesnel-Vallée A Morgan SP Missing the Target? Correspondence of Fertility Intentions and Behavior in the U.S. Population Research and Policy Review. 2003;22:497-525. 10.1023/B:POPU.0000021074.33415.c1

[10] Hunan Provincial Bureau of Statistics. HUNAN STATISTICAL YEARBOOK, 2021. Available: http://222.240.193.190/2021tjnj/indexch.htm. Accessed: 20 October 2022.

[11] Wells YO Dietsch E Childbearing traditions of Indian women at home and abroad: An integrative literature review. Women Birth. 2014;27:e1-6. 10.1016/j.wombi.2014.08.006 25257377

[12] Briefng C. China Releases Supporting Measures for Three–Child Policy 2021 [updated 2021-03-28]. Available: https://www.china-briefing.com/news/china-releases-supporting-measures-for-three-child-policy/. Accessed: 20 October 2022.

[13] Yang Y He R Zhang N Li L Second-Child Fertility Intentions among Urban Women in China: A Systematic Review and Meta-Analysis. Int J Environ Res Public Health. 2023;20:3744. 10.3390/ijerph20043744 36834437 PMC9962327

[14] May-Panloup P Boucret L Chao de la Barca JM Desquiret-Dumas V Ferré-L’Hotellier V Morinière C Ovarian ageing: the role of mitochondria in oocytes and follicles. Hum Reprod Update. 2016;22:725-43. 10.1093/humupd/dmw028 27562289

[15] Jing W Liu J Ma Q Zhang S Li Y Liu M Fertility intentions to have a second or third child under China’s three-child policy: a national cross-sectional study. Hum Reprod. 2022;37:1907-18. 10.1093/humrep/deac101 35554542

[16] Zhu C Yan L Wang Y Ji S Zhang Y Zhang J Fertility Intention and Related Factors for Having a Second or Third Child Among Childbearing Couples in Shanghai, China. Front Public Health. 2022;10:879672. 10.3389/fpubh.2022.879672 35757654 PMC9218102

[17] Yan Z Hui L Wenbin J Liuxue L Yuemei L Bohan L Third birth intention of the childbearing-age population in mainland China and sociodemographic differences: a cross-sectional survey. BMC Public Health. 2021;21:2280. 10.1186/s12889-021-12338-8 34906129 PMC8670058

[18] Ning N Tang J Huang Y Tan X Lin Q Sun M Fertility Intention to Have a Third Child in China following the Three-Child Policy: A Cross-Sectional Study. Int J Environ Res Public Health. 2022;19:15412. 10.3390/ijerph192215412 36430129 PMC9690853

[19] Aassve A Cavalli N Mencarini L Plach S Livi Bacci M The COVID-19 pandemic and human fertility. Science. 2020;369:370-1. 10.1126/science.abc9520 32703862

[20] Guo W Tan Y Yin X Sun Z Impact of PM(2.5) on Second Birth Intentions of China’s Floating Population in a Low Fertility Context. Int J Environ Res Public Health. 2019;16:4293. 10.3390/ijerph16214293 31694255 PMC6862601

[21] Odusina EK Ayotunde T Kunnuji M Ononokpono DN Bishwajit G Yaya S Fertility preferences among couples in Nigeria: a cross sectional study. Reprod Health. 2020;17:92. 10.1186/s12978-020-00940-9 32527271 PMC7291488

[22] Zhou Y The Dual Demands: Gender Equity and Fertility Intentions After the One-Child Policy. J Contemp China. 2018;28:367-84. 10.1080/10670564.2018.1542219 31440018 PMC6706249

[23] Liu J Zhou Z Mothers’ Subjective Well-Being after Having a Second Child in Current China: A Case Study of Xi’an City. Int J Environ Res Public Health. 2019;16:3823. 10.3390/ijerph16203823 31658744 PMC6843609

[24] Qian Y Liu XY Fang B Zhang F Gao R Investigating Fertility Intentions for a Second Child in Contemporary China Based on User-Generated Content. Int J Environ Res Public Health. 2020;17:3905. 10.3390/ijerph17113905 32486446 PMC7312720

[25] Hammarberg K Collins V Holden C Young K McLachlan R Men’s knowledge, attitudes and behaviours relating to fertility. Hum Reprod Update. 2017;23:458-80. 10.1093/humupd/dmx005 28333354

[26] Cheng PJ Duan T China’s new two-child policy: maternity care in the new multiparous era. BJOG. 2016;123 Suppl 3:7-9. 10.1111/1471-0528.14290 27627588

[27] WU-B-x TANG -J. Second fertility intention and its influencing factors among women aged 35-50 years under universal two-child policy. Chin J Publ Health. 2019;35:1685-689.

[28] Zhang J Tian Y Housework Division and Second-Child Fertility Anxiety among Couples in China: The Urban and Rural Differences. Int J Environ Res Public Health. 2019;16:3910. 10.3390/ijerph16203910 31618873 PMC6843918

[29] Bongaarts J Completing the fertility transition in the developing world: The role of educational differences and fertility preferences. Popul Stud (Camb). 2003;57:321-35. 10.1080/0032472032000137835 14602532

[30] Liu P Cao J Nie W Wang X Tian Y Ma C The Influence of Internet Usage Frequency on Women’s Fertility Intentions-The Mediating Effects of Gender Role Attitudes. Int J Environ Res Public Health. 2021;18:4784. 10.3390/ijerph18094784 33946141 PMC8124929

[31] Zheng Y Yuan J Xu T Chen M Liang H Connor D Socioeconomic status and fertility intentions among Chinese women with one child. Hum Fertil (Camb). 2016;19:43-7. 10.3109/14647273.2016.1154988 27006090

[32] Chu K Zhu R Zhang Y Pang W Feng X Wang X Fertility Intention Among Chinese Reproductive Couples During the COVID-19 Outbreak: A Cross-Sectional Study. Front Public Health. 2022;10:903183. 10.3389/fpubh.2022.903183 35801249 PMC9253424

[33] Bauer G Kneip T Fertility From a Couple Perspective: A Test of Competing Decision Rules on Proceptive Behaviour. Eur Sociol Rev. 2013;29:535-48. 10.1093/esr/jcr095

[34] Stein P Pavetic M A nonlinear simultaneous probit-model for the investigation of decision-making processes: modelling the process of setting up a family in partnerships. Qual Quant. 2013;47:1717-32. 10.1007/s11135-011-9622-y

[35] Mencarini L Vignoli D Zeydanli T Kim J Life satisfaction favors reproduction. The universal positive effect of life satisfaction on childbearing in contemporary low fertility countries. PLoS One. 2018;13:e0206202. 10.1371/journal.pone.0206202 30517115 PMC6281189

[36] Dribe M Hacker JD Scalone F The impact of socio-economic status on net fertility during the historical fertility decline: a comparative analysis of Canada, Iceland, Sweden, Norway, and the USA. Popul Stud (Camb). 2014;68:135-49. 10.1080/00324728.2014.889741 24684711 PMC4244229

[37] Zeng Y Hesketh T The effects of China’s universal two-child policy. Lancet. 2016;388:1930-8. 10.1016/S0140-6736(16)31405-2 27751400 PMC5944611

