# Supplementary material for: Fertility intentions to have a second or third child among the childbearing-age population in Central China under China’s three-child policy: A cross-sectional study
Source: J Glob Health. 2023 Jul 14;13:04072. doi: 10.7189/jogh.13.04072 (PMC10345887; doi:10.7189/jogh.13.04072)
Supplement: Online Supplementary Document [file jogh-13-04072-s001.pdf]

Fertility intentions to have a second or third child among the childbearing-age population in Central China under China's three-child policy: a cross-sectional study

Online Supplementary Document

Table S1 The prevalence of age-specified fertility intentions by family type

Table S2 Multivariate logistic regression of one child family fertility intention

Table S3. Multivariate logistic regression of two child family fertility intention

Table S1. The prevalence of age-specified fertility intentions by family type

Family type	Intention to have another child		No Intention to have another child	
	n (%)	95% CI*	n (%)	95% CI*
**One-child family**				
20-24	95(42.60)	36.06-49.14	128(57.40)	50.86-63.94
25-29	374(37.18)	34.19-40.17	632(62.82)	59.83-65.81
30-34	578(32.88)	30.68-35.08	1180(67.12)	64.92-69.32
35-39	254(28.13)	25.19-31.07	649(71.87)	68.93-74.81
40-44	89(17.80)	14.44-21.16	411(82.20)	78.84-85.56
45-49	54(11.51)	8.61-14.41	415(88.49)	85.59-91.39
**Two-child family**				
20-24	25(24.27)	15.85-32.69	78(75.73）	67.31-84.15
25-29	86(14.68)	11.80-17.55	500(85.32)	82.45-88.20
30-34	311(12.84)	11.51-14.17	2111(87.16)	85.83-88.49
35-39	187(8.80)	7.59-10.00	1939(91.20)	90.00-92.41
40-44	100(8.01)	6.50-9.51	1149(91.99)	90.49-93.50
45-49	41(7.19)	5.07-9.32	529(92.81)	90.68-94.93

****95% CI** – 95% Confidence interval

Table S2. Multivariate logistic regression of one child family fertility intention

Factors	One child family fertility intention	
	**Multivariate model aOR* (95% CI†)**	***P* value**
**The gender of the respondents**		
Women	1 (reference)	
Men	1.33(1.00-1.78)	0.051
**Age(years)**		
20-24	1 (reference)	
25-29	1.06(0.78-1.45)	0.691
30-34	1.05(0.77-1.42)	0.766
35-39	0.87(0.63-1.21)	0.441
40-44	**0.49(0.34-0.71)**	**<0.001****
45-49	**0.27(0.18-0.41)**	**<0.001****
**Residence**		
Urban	1 (reference)	
Rural	**1.33(1.15-1.54)**	**<0.001****
**Marital status**		
First marriage	1 (reference)	
Remarriage	**1.86(1.11-3.11)**	**0.019‖**
**Educational level**		
Junior high school or below	1 (reference)	
Senior high school or equivalent	**0.76(0.60-0.96)**	**0.023‖**
College or higher	**0.62(0.50-0.78)**	**<0.001****
**Average working hours**		
<7 hours	1 (reference)	
7-12 hours	**0.81(0.70-0.94)**	**0.005¶**
>12 hours	**0.65(0.44-0.97)**	**0.033‖**
**Floating population**		
No	1 (reference)	
Yes	1.07(0.93-1.24)	0.348
**Living with parents**		
No	1 (reference)	
Yes	1.12(0.97-1.29)	0.126
**Childcare support**		
No	1 (reference)	
Yes	**1.60(1.37-1.88)**	**<0.001****
**Relationship between mother-in-law and daughter-in-law**		
Bad	1 (reference)	

Ordinary	1.30(0.81-2.08)	0.281
Good	**1.93(1.20-3.09)**	**0.006¶**
**Marital satisfaction**		
Dissatisfied	1 (reference)	
Satisfied	**1.84(1.35-2.51)**	**<0.001****
**Children's educational barriers‡**		
Yes	1 (reference)	
No	**1.28(1.03-1.580)**	**0.024‖**
**Children's medical barriers§**		
Yes	1 (reference)	
No	1.14(0.92-1.41)	0.233
**Health condition of both spouses**		
Both healthy	1 (reference)	
Both unhealthy	**0.74(0.56-0.98)**	**0.033‖**
One of only healthy	0.90(0.70-1.14)	0.376
**Health condition of both parents**		
Both healthy	1 (reference)	
Both unhealthy	1.24(0.58-2.64)	0.577
One of only healthy	0.98(0.72-1.33)	0.896
**Loan situation**		
Yes	1 (reference)	
No	**1.22(1.06-1.41)**	**0.005¶**
**Size of living house**		
<100 square meters	1 (reference)	
100-200 square meters	**1.22(1.07-1.41)**	**0.004¶**
>200 square meters	**2.01(1.37-2.96)**	**<0.001****
**Financial situation of parents**		
Poor	1 (reference)	
Rich	1.02(0.85-1.22)	0.859
**The gender pattern of one-child family**		
Boy	1 (reference)	
Girl	**1.24(1.09-1.41)**	**0.001¶**

***aOR** – adjusted odds ratio; †**95% CI** – 95% Confidence interval

‡**Children's educational barriers** = The stress of the accessibility of educational resources and meet the educational needs of children.

§**Children's medical barriers** = The stress of the accessibility of medical resources and meet the health needs of children.

‖*P* value < 0.05

¶*P* value < 0.01

****P* value < 0.001**

Table S3. Multivariate logistic regression of two child family fertility intention

Factors	Two child family fertility intention	
	Multivariate model aOR* (95% CI†)	*P* value
**The gender of the respondents**		
Women	1 (reference)	
Men	**1.98(1.42-2.76)**	**<0.001****
**Age(years)**		
20-24	1 (reference)	
25-29	0.73(0.43-1.24)	0.237
30-34	0.65(0.40-1.07)	0.089
35-39	**0.43(0.26-0.71)**	**0.001¶**
40-44	**0.38(0.22-0.65)**	**<0.001****
45-49	**0.30(0.16-0.53)**	**<0.001****
**Residence**		
Urban	1 (reference)	
Rural	1.13(0.93-1.39)	0.223
**Marital status**		
First marriage	1 (reference)	
Remarriage	**4.29(2.83-6.49)**	**<0.001****
**Educational level**		
Junior high school or below	1 (reference)	
Senior high school or equivalent	0.87(0.70-1.07）	0.174
College or higher	**0.62(0.50-0.78)**	**<0.001****
**Average working hours**		
<7 hours	1 (reference)	
7-12 hours	**0.78(0.65-0.92)**	**0.004¶**
>12 hours	0.87(0.57-1.30)	0.508
**Insurance**		
No	1 (reference)	
Yes	**0.57(0.36-0.91)**	**0.018‖**
**Parent's family situation**		
Both only child	1 (reference)	
Both non-only child	1.67(0.98-2.86)	0.061
One of only child	1.21(0.70-2.12)	0.493
**Living with parents**		
No	1 (reference)	
Yes	1.06(0.88-1.27)	0.530
**Childcare support**		
No	1 (reference)	
Yes	**1.46(1.21-1.76)**	**<0.001****
**Relationship between mother-in-law and daughter-in-law**		
Bad	1 (reference)	
Ordinary	0.99(0.53-1.86)	0.985

Good	1.06(0.57-1.97)	0.864
**Marital satisfaction**		
Dissatisfied	1 (reference)	
Satisfied	**2.84(1.77-4.56)**	**<0.001****
**Children's educational barriers‡**		
Yes	1 (reference)	
No	**1.35(1.03-1.76)**	**0.027‖**
**Children's medical barriers§**		
Yes	1 (reference)	
No	1.13(0.86-1.49)	0.373
**Health condition of both spouses**		
Both healthy	1 (reference)	
Both unhealthy	0.74(0.53-1.03)	0.078
One of only healthy	**0.57(0.39-0.82)**	**0.003¶**
**Loan situation**		
Yes	1 (reference)	
No	1.15(0.98-1.36)	0.093
**Size of living house**		
<100 square meters	1 (reference)	
100-200 square meters	**1.21(1.01-1.44)**	**0.038‖**
>200 square meters	**1.86(1.32-2.61)**	**<0.001****
**Financial situation of parents**		
Poor	1 (reference)	
Rich	1.11(0.90-1.36)	0.336
**The gender pattern of two-child family**		
Girl and boy	1 (reference)	
Boy and boy	**1.27(1.02-1.59)**	**0.035‖**
Boy and girl	0.89(0.70-1.13)	0.348
Girl and girl	**2.76(2.23-3.42)**	**<0.001****

***aOR** = adjusted odds ratio; †**95% CI** = 95% Confidence interval

‡**Children's educational barriers =** The stress of the accessibility of educational resources and meet the educational needs of children.

§**Children's medical barriers =** The stress of the accessibility of medical resources and meet the health needs of children.

‖***P* value < 0.05**

¶***P* value < 0.01**

****P* value < 0.001**
